# Feasibility of novel approaches to detect viable *Mycobacterium tuberculosis* within the spectrum of the tuberculosis disease

**DOI:** 10.3389/fmed.2022.965359

**Published:** 2022-08-22

**Authors:** Sogol Alebouyeh, Brian Weinrick, Jacqueline M. Achkar, Maria J. García, Rafael Prados-Rosales

**Affiliations:** ^1^Department of Preventive Medicine and Public Health and Microbiology, Autonoma University of Madrid, Madrid, Spain; ^2^Trudeau Institute, Saranac Lake, NY, United States; ^3^Departments of Medicine, Microbiology and Immunology, Albert Einstein College of Medicine, Bronx, NY, United States

**Keywords:** *Mycobacterium tuberculosis*, LTBI (Latent TB infection), TB spectrum, viable bacteria, subclinical TB, incipient TB

## Abstract

Tuberculosis (TB) is a global disease caused by *Mycobacterium tuberculosis* (*Mtb*) and is manifested as a continuum spectrum of infectious states. Both, the most common and clinically asymptomatic latent tuberculosis infection (LTBI), and the symptomatic disease, active tuberculosis (TB), are at opposite ends of the spectrum. Such binary classification is insufficient to describe the existing clinical heterogeneity, which includes incipient and subclinical TB. The absence of clinically TB-related symptoms and the extremely low bacterial burden are features shared by LTBI, incipient and subclinical TB states. In addition, diagnosis relies on cytokine release after antigenic T cell stimulation, yet several studies have shown that a high proportion of individuals with immunoreactivity never developed disease, suggesting that they were no longer infected. LTBI is estimated to affect to approximately one fourth of the human population and, according to WHO data, reactivation of LTBI is the main responsible of TB cases in developed countries. Assuming the drawbacks associated to the current diagnostic tests at this part of the disease spectrum, properly assessing individuals at real risk of developing TB is a major need. Further, it would help to efficiently design preventive treatment. This quest would be achievable if information about bacterial viability during human silent *Mtb* infection could be determined. Here, we have evaluated the feasibility of new approaches to detect viable bacilli across the full spectrum of TB disease. We focused on methods that specifically can measure host-independent parameters relying on the viability of *Mtb* either by its direct or indirect detection.

## Introduction

The outcome of *Mycobacterium tuberculosis* (*Mtb*) infection in humans results in a continuum of infectious states. While there are many nuances, the clinically asymptomatic latent tuberculosis infection (LTBI), states the symptomatic disease, active tuberculosis (TB) are the most common and at opposite ends of the spectrum. Decades of clinical observation and recent confirmation with more sensitive imaging approaches prompted clinical researchers to propose both incipient TB and subclinical TB as additional early disease states between LTBI and TB [reviewed in (1)]. However, the relationship between LTBI and TB among those in clinical practice, which is based on longitudinal studies with long-term follow-up, all epidemiologic data, establishes that: (a) active TB cases represent about 5% of initial infections and; (b) the remaining 95% of LTBI individuals who probably will never develop the disease account for the major reservoir of *Mtb* worldwide; (c) transitioning from LTBI to TB (progression) occurs in about 5–10% of the cases and requires perturbation of the immune system, which under normal circumstances controls bacterial replication (2–4). Because reactivation of LTBI leads to a contagious state, enormous efforts are directed to diagnosing and treating LTBI. Accordingly, WHO considers the control of LTBI as crucial milestone for TB elimination within its current *End TB Strategy* (5). It is therefore reasonable to concentrate efforts in the determination of bacterial presence around early TB statuses where no clinical signs are detected to assess the likelihood of potential transition to advanced stages of the disease. However, limitations imposed by the extremely low bacterial burden make this quest very challenging.

Here, we have evaluated the potential contribution of new approaches that could greatly impact the detection of viable *Mtb* in the context of individuals undergoing early stages of the disease including incipient TB and subclinical TB.

## The very cryptic concept of latency in tuberculosis

By definition, the state of latency occurs when a microorganism establishes itself within the host in a dormant or silent state without perturbing homeostasis nor inducing clinical symptoms or disease (6). Several microorganisms have the ability to develop latency within the carrying host and the mechanisms governing this phenomenon will depend on the nature of both the microbe and the host. Although *Mtb* is a classic example of a microorganism which can establish latency for long periods of time, the underlying pathogenesis and immune responses around it are incompletely understood. A recent study in *Mtb*-infected cynomolgus macaques shows that latency spans multiple granuloma environments with divergent responses within a single animal as well as across several animals, ranging from uncontrolled bacterial replication to resolved and cleared infection (7). To make the picture more complex, the presence of different *Mtb* strains has been recently identified within the same individual and even within the same granuloma from TB patients (8). Whether this scenario could exist in latently infected individuals is currently unknown. It is possible that LTBI likely consists of a heterogeneous spectrum of infections more than a single status. In other words, an individual with LTBI could carry granulomas, each one being a localized microenvironment with different degrees of progression and magnitude of the localized immune response. Moreover, recent reports indicate that dormant *Mtb* can also be found in other non-granulomatous locations, such as bone marrow (9) or adipose tissue (10), highlighting the complexity of LTBI.

## It is not all about latent tuberculosis infection and tuberculosis

Decades of clinical observations show that LTBI and TB are both at opposite ends of a continuous spectrum of *Mtb* infection leading to different levels of TB pathophysiological manifestations (1, 11). In this context, both incipient TB and subclinical TB share the feature with LTBI that the person who is under these stages carries viable *Mtb*. However, while in LTBI a quiescent state for *Mtb* is considered, metabolic activity is thought to be higher as a surrogate of ongoing infection in both incipient and subclinical states. Incipient TB was proposed to describe the “constellation of upper lobe opacities over 2 cm^2^ in size, not attributable to another disease and occurring in an asymptomatic, apparently immunocompetent host with prior TB exposure” (1), and establishes that a metabolically active bacillus can increase the chances to progress to active infection in the absence of clinical symptoms. The concept of subclinical TB was proposed in the context HIV-TB coinfection and refers to a status where there are “microbiologic and/or radiographic evidence of pulmonary TB in an asymptomatic immunocompromised host” (1). This expansion of the clinical categories could help to understand dynamics of the disease. Moreover, it could help to diversify and stratify the potential interventions and to prevent progression from LTBI to TB (2–4). Nevertheless, more studies are needed to uncover the status of the bacillus out of LTBI or active TB states.

Overall, realization of such heterogeneity around TB infection is important because it could enable clinicians to better evaluate the risk of reactivation, thus prioritize preventive treatment (12). Consequently, the presence of viable mycobacteria in a TB infected individual will represent a major contributor to the risk of reactivation. The question is at what level of the spectrum would it be possible to detect viable bacteria and whether we have diagnostic tools to do so.

## What do we know about the status of *Mtb*?

One of the main reasons why we have not generated the necessary effective tools to enhance the control of TB disease is our incomplete understanding of the mycobacterial physiology during latency, which is closely related with the fact that it is extremely difficult to study bacterial physiology during human asymptomatic infection. The challenge of obtaining viable *Mtb* from a latently infected individual includes the need of a biopsy of a granuloma, which would be not feasible given the very small size and the required invasive procedure. Studies on macaques have shown a higher proportion of sterile granulomas in animals carrying LTBI, suggesting the participation of other niches in the reactivation of the disease. Moreover, the risk of reactivation was associated with inflammation rather than with number of granulomas (13). In addition, dormant *Mtb* typically features a lack of responsiveness to acid-fast staining, the development of drug tolerance and a viable but non-culturable (VBNC) phenotype (14), also named differentially culturable tubercle Mtb (DCTB) (15). The key question is, what is the evidence for Mtb being viable during silent infection? Some studies have used the mutation rate as a “molecular clock” (16) to estimate the generation time of *Mtb* during longitudinal studies in LTBI subjects. While those performed in cynomolgus macaques estimated that *Mtb* isolated from lesions obtained from LTBI and reactivated animals (TB) show the same mutational rate (17), studies using samples from TB patients determined that *Mtb* manifests a reduced replication rate during latency relative to TB (18). A more recent study analyzing initial infection and progression of TB patients to LTBI or TB confirmed that mutation rates are sustained at a high level up to 2 years after infection. After this period of “early latency,” *Mtb* switch to a state with an increased generation time (“late latency”) (19). Importantly, the results from this study imply that TB has a shorter incubation period than previously thought and are consistent with a recent longitudinal analysis of the natural history of TB, where a median incubation time of months to 2 years was determined (20). Further, TB reactivation rates dramatically decline after this maximum period of 2 years.

There are no studies focused on understanding the status of the bacillus during incipient or subclinical TB. Nevertheless, the determination of the status of *Mtb* in either early or late latency, as well as in incipient or subclinical states, should include the detection of viable bacteria. A bacterium is considered viable when it can resume cellular activity while in a dormant state and do not cross a “point of no return,” thus being able to resuscitate from latent infection. That “no return” point has been assigned to the moment in which no metabolic activity is detected regardless bacterial envelope integrity (21). In this respect, it has been previously established that non-replicating, considered dormant, *Mtb* are metabolically active (2, 22). Such metabolic activity is supposed to be reduced to the minimum during latency and increase further in incipient and subclinical TB statuses.

## Limited performance of current diagnostic approaches in asymptomatic tuberculosis infected individuals

While diagnosis of TB is linked to clinical symptoms such as fever, chronic cough, weight loss or hemoptysis together with the microbiological identification of *Mtb* in clinical samples, the diagnosis of other states of the disease, and particularly LTBI, are more problematic. Most of the tests directed to detect *Mtb* or its products have been efficiently implemented in the context of TB, on the contrary, there is no gold standard test for LTBI diagnosis. The gold-standard procedures for detection of viable bacilli are the culture-dependent techniques (23), yet they are unable to detect VBNC bacteria. Importantly, this VBNC bacterial population, which arises associated with stresses or latency, would be characterized as dead when using solid media or standard liquid media as a method for enumeration (24). Of note, studies have shown that certain stimuli, such as murolytic enzymes, can make part of the VBNC population resume growth (25, 26), indicating the potential infectivity of those samples falsely declared TB-negative. The challenge of VBNC cells clearly limits the power of culture-based approaches to detect viable *Mtb*. Alternatively, some non-culture-dependent procedures aimed at detecting viable bacteria are based on bacterial cell membrane integrity and the use of dyes that only penetrate compromised or damaged cell envelopes [see for revision (24)]. One limitation of such approaches is their lack of specificity for *Mtb*, and an inability to reliably detect viable *Mtb* in complex materials such as clinical samples. In addition, a confounding factor for these procedures is the demonstrated cell envelope alteration that *Mtb* undergoes while dormant making it lose their characteristic acid-fast staining (27). The method to diagnose TB and rifampicin resistance recommended by the World Health Organization (WHO), the Xpert MTB/RIF, is based on the detection of *Mtb* DNA in a clinical specimen, yet it is unable to discern between live and dead bacilli. Other methods include detection of cell wall-derived lipoarabinomannan (LAM) in urine or antigen 85 (Ag85) protein (28), as well as several *Mtb*-specific small molecules (29). The urinary LAM test is recommended despite it provides moderate sensitivity (30). Importantly, this assay measures a labile *Mtb*-derived molecule and its readout could be linked to bacterial viability. Nevertheless, the residence time of LAM in fluidic samples and its connection with bacteria actively producing it has never been determined in human. Moreover, specificity issues related to the presence of these products in infected individuals harboring either *Mtb* or non-tuberculous mycobacteria (NTM) might reduce the expectations around them in assessing TB.

By definition, LTBI is “a state of persistent immune response to prior acquired *Mycobacterium tuberculosis* antigens without evidence of clinically manifested active TB” (31). Accordingly, diagnosis of LTBI is established through measurement of the host immune response to *Mtb* antigens, either by tuberculin skin test (TST) or variations of the interferon gamma release assay (IGRA) [see for revision (11)]. It is assumed that such immune response is a consequence of the presence of viable bacteria. However, recent epidemiological analysis of the natural history of individuals with TB immunoreactivity submitted to therapeutic or immunosuppressive treatments indicates that a surprisingly low fraction of people (1–11%) developed disease. This suggests that most LTBI individuals were no longer infected, either because the bacillus was not viable or had lost its pathogenic potential (32). This analysis has important consequences for the way LTBI is defined, implying that the current available LTBI tests lack the capacity to predict who is at higher risk of progression to TB (2) and are unable to distinguish chronic infection from persistent immunological memory after presumed bacterial elimination. Moreover, the readout of these tests is not related with the presence of the bacillus but based on the assumption that the measured immune responses allow the control of dormant bacilli. A recent report on the host transcriptional response to TB preventive therapy in a small cohort of patients showed that the whole blood transcriptional profile in a large fraction of IGRA+ individuals clustered with the IGRA- individuals’ profile, indicating that some individuals diagnosed as LTBI-positive may not carry viable bacilli and will never progress to active TB disease through reactivation (33). Overall, these studies suggest that preventive treatment for LTBI will be strongly improved if the detection of viable bacilli is accomplished for these individuals. While the combined application of microbiological approaches, chest radiography, and Xpert MTB/RIF allows diagnostic of subclinical TB, there are no approved diagnostic tests for incipient TB at the present time.

An important variable to consider in individuals with latent, incipient, or subclinical TB at the time of microbiological diagnosis is the test sample. Typically, these individuals do not generate sufficient or any sputum due to the absence symptomatic coughs. This problem is shared with pediatric and HIV-infected patients with active TB, prompting the consideration of alternative sample types (33, 34). Saliva, blood, urine, stool, gastric and bronchoalveolar lavage fluids, fine needle aspirates, and exhaled breath condensate have all been evaluated as alternative samples for tuberculosis diagnosis (35). In addition, the very low bacterial burden at this side of the TB spectrum typically influences strategies for the design of novel approaches based on the host immune response. However, as mentioned above, approaches aimed at the detection of viable *Mtb* in LTBI and other early and asymptomatic infection stages of the disease would be key to efficiently direct therapies.

## Approaches to detect viable mycobacteria

There are several approaches that could potentially contribute to the improvement of the diagnosis through the detection of mycobacterial viability linked to both host-independent and *Mtb*-specific processes.

### Detection of released MPT64

**FIGURE 1:**
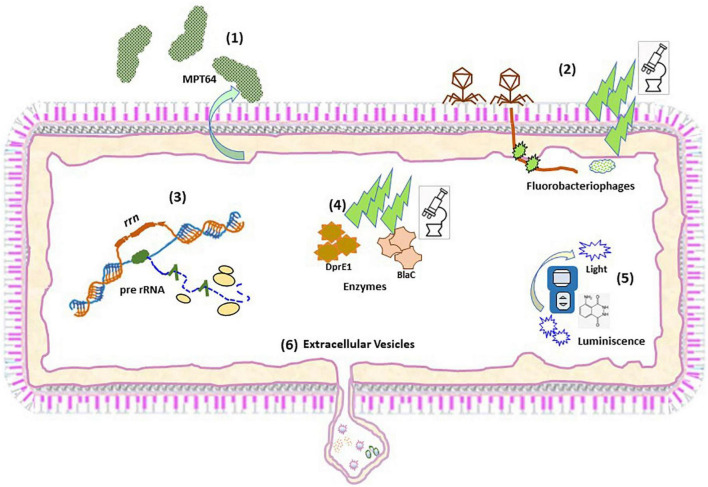
Schematic representation of the different approaches to detect viable *Mtb*. Schematic representation of the procedures to detect: MTP64 **(1)**; Fluorobacteriophages **(2)**; pre-rRNA **(3)**; Fluorescent activity probes **(4)**; Chemilumiscent probes **(5)**; and Extracellular vesicles **(6)**.

**FIGURE 2:**
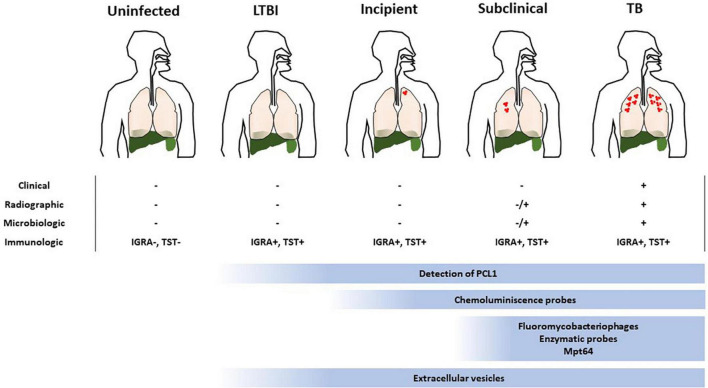
Suitability of the different approaches to detect viable Mtb within the TB disease spectrum. TB is represented as a spectrum of different states where current immunologic approaches to diagnose it are confounding. Determination of the viability of the infecting bacillus is key to ascertain the risk of developing TB, specially at early stages of the disease (LTBI, incipient and subclinical TB). The low bacterial burden associated to such states makes it difficult to implement methods to accomplish this. The suitability of the current methods to detect viable *Mtb* are depicted and the range where they could be useful is indicated.

Very recently, a culture free assay has been developed based on the ability of *Mtb* to release the antigen MPT64 when the sputum sample is gently heated (46^°^C) (36). Subsequent detection of MPT64 via enzyme-linked immunosorbent assay (ELISA) allows diagnosis within 5 h (Figure 1). Considering that only live *Mtb* is able to do this and that this protein is absent from NTM, this assay represents a promising development in TB diagnosis. MPT64 seems a useful biomarker for detection of viable bacilli. Nevertheless, previous transcriptomic studies showed downregulation of MPT64 (Rv1980c) in several *in vitro* dormancy models (37, 38). Therefore, the suitability of MPT64 as biomarker to detect dormant bacilli remains to be established. Nevertheless, it would be difficult to implement this approach in latent TB or incipient TB where very few bacilli may be present. Consequently, its use might be suitable for advanced stages of the disease such as TB or some forms of subclinical TB (Figure 2 and Table 1).

**TABLE 1:** Approaches to detect viable bacilli in the context of Tuberculosis disease.

Approaches	Technical requirements	Physical detection of mycobacteria	Clinical samples tested	Detection limit	Detected VBNC bacilli	Suitability on TB spectrum^$^
MPT64 released	ELISA	No	Sputum	3.3 × 10^2^ bacilli/ml	N.D.^#^	Subclinical-Active
Fluoromycobacteriophages	Fluorescence microscopy or flow cytometry	Yes	Sputum	<10^4^ bacilli/ml	INH-persisters	Subclinical-Active
Pre-rRNA	Quantitative RT-PCR	No	Sputum, BAL, Adenopathy, Fluids, Biopsies	1 pg/μl (culture negative sputum sample)	Culture negative clinical samples TB patients	Latent-Incipient-Subclinical-Active
Fluorescent activity probes	Fluorescence microscopy or flow cytometry	Yes	Sputum	10^2^ bacilli/sample	*In vitro* cultures	Subclinical-Active
Chemiluminescent probes	Luminometer	Yes	Sputum	4 × 10^3^ bacilli/sample	*In vitro* cultures	Incipient-Subclinical-Active
Extracellular vesicles	Filtration/Immunocapture	No	Plasma, Urine	4.5 × 10^6^ EV/ml*	N.D.^#^	Latent-Incipient-Subclinical-Active

^$^Hypothetical suitability of the approaches to detect bacilli along the TB spectrum statuses.

^#^N.D., not determined.

*Measured in a lateral flow immune ELISA using exosome-specific antibodies combined with silver nanoparticles on plasma samples (70).

Biopsies: colon, liver; Fluids: blood, cerebrum spinal fluid, urine, pleural, synovial.

### Fluoromycobacteriophages

Some approaches have exploited genetically modified thermosensitive mycobacteriophages carrying fluorescent reporter genes, which after infection of metabolically active mycobacterial cells transcribe and translate the fluorescence gene and the signal can be readily detected by fluorescence microscopy or flow cytometry (39, 40) (Figure 1). The ϕ^2^GFP10 fluorophage allowed the visualization of individual *Mtb* bacilli in clinical sputum samples down to paucibacillary concentrations (<10^4^ bacilli/ml) (41, 42). In a different report, mCherrybombϕ was evaluated for detection of *Mtb* and rifampicin resistance in sputum samples from presumptive TB patients, achieving detection in less than 5 days (43, 44). In the former approach, experimental setups combining drug treatment including isoniazid and phage showed a dramatic reduction in average fluorescence, yet a small fraction of cells retained maximal signal, indicating that this approach can target persister cells (41). One important consideration when developing mycobacteriophages for detection of viable *Mtb* is the phage-host specificity. Although the genetic bases of such specificity are largely unknown, it may be possible to find mycobacteriophages, which bind preferentially to *Mtb* over other mycobacteria (45). However, a recent study suggests that such specificity is not due to inability of the phage to infect NTM, rather unknown mechanisms of resistance to plaque formation may be responsible for the observed differences between *Mtb* and NTM (46). Furthermore, the identification of mycobacteriophages receptors restricted to *Mtb* will be essential to understand the specificity of this interaction (47). In combination with genetic engineering aimed at restricting the host range of such phages to *Mtb*, although able to infect a wide range of strains, mycobacteriophages could represent a great alternative for the identification of live *Mtb* within clinical samples. Although sensitive, fluorobacteriophages appear to be suitable for sputum samples. Due to a reduced bacillary load associated to early infection statuses close to LTBI this approach would only be effective around TB or subclinical TB (Figure 2 and Table 1).

### Detection of pre-rRNA

Considering the essential function performed by ribosomes in the basic metabolic activity of bacteria, the active synthesis of ribosomal RNA is critical to maintain bacterial viability (22), illustrated by the dependence of resuscitation on ribosomal activity (48). Detection of bacterial ribosomal RNA (rRNA) synthesis is linked to the occurrence of a basal metabolic activity, regardless of the number or kind of genes expressed in a particular condition. Synthesis of rRNA is made through the synthesis of a long product (pre-rRNA) after RNA polymerase reads the ribosomal operon sequence (*rrn*). Leader and tail fragments are further removed by digestion by RNAses to give the 3 components of mature rRNA (16S, 23S, and 5S) (Figure 1). The mature rRNA links afterward to ribosomal proteins to form ribosomes. The high level of mature mRNA inside bacterial cells makes it a good target allowing a low limit of detection. Mature rRNA is less stable than DNA but more stable than pre-rRNA because it is complexed with proteins. Therefore, contrary to pre-rRNA, detection of DNA (11) or mature rRNA (Xpert MTB) does not accurately represent bacterial viability (48). However, the detection of mature rRNA by semiquantitative PCR has been used as a putative marker of *Mtb* viability in the context of bacterial susceptibility and therapeutic efficacy (49). Beside instability, another feature of pre-rRNA processed region it is that it contains species-specific sequences due to its higher variability compared to mature rRNA, making its detection a good biomarker candidate to identify viable bacteria in a complex sample. Species-specific detection of pre-rRNA by RT-qPCR has been frequently used to differentiate viable from dead bacterial cells in drinking water samples and in serum samples (50).

In mycobacteria, the sequences of pre-rRNA are also species-specific and, when detected in a culture negative sample, it is inferred that the culture contains viable bacteria. Using RT-qPCR we were able to detect viable bacteria in MGIT negative tubes, thus indicating putative presence of VBNC *Mtb* (unpublished results). Moreover, in our previous study we detected *Mtb* pre-rRNA in pulmonary and extrapulmonary culture negative human clinical samples at very low concentration (1 pg/μl cDNA) by using RT-qPCR (51) (Table 1). Remarkably, the positivity found corresponded to about 17% of those culture negative samples, showing not only that viable bacilli could be detected in the absence of growth but also suggesting the suitability of the method in the context of LTBI. Of public health relevance, those previous samples turn out to be diagnosed TB negative by using standard procedures. More recently, that target was applied to test sterilizing activity of drugs and accelerate the measurement of *in vitro* drug activity in *Mtb* (52). The basic physiological function provided by pre-rRNA makes this bacterial component a robust biomarker to detect viable bacilli, regardless their growing condition or VBNC phenotype. Moreover, due to its relationships with the basic metabolic activity of the bacillus, the detection of pre-rRNA could readily correlates from a minimum level during LTBI to progressively increase levels in incipient and subclinical infection. Therefore, this approach is suitable to identify *Mtb* bacilli in all stages of the TB disease spectrum (Figure 2 and Table 1).

### Fluorescence probes

As previously mentioned, one of the main limitations in the use of fluorochromes to detect *Mtb* is that most of them do not discriminate between live and dead bacteria. One example of this is auramine O, which stains acid-fast microorganisms and binds to cell wall-associated mycolic acids in mycobacteria (53). Moreover, this lack of specificity for *Mtb* is extended to other fluorochromes targeting different mycobacterial components such as peptidoglycan motifs (54). One approach that has shown promise is the development of probes revealing the activity of *Mtb*-specific enzymes on fluorogenic substrates such as those for trehalose mycolyltransferases (55) or sulfatases (56). This approach has been advanced through the development of a cephalosporin-derived fluorophore with specificity for BlaC, the main β-lactamase produced by *Mtb* (57). The probe could achieve the detection and imaging of less than 100 bacilli in unprocessed sputum samples with very high specificity (58). A breakthrough in the detection of single live *Mtb* has been recently accomplished by developing a fluorescence probe, which recognizes both BlaC and the enzyme decaprenylphosphoryl-β-d-ribose 2′-epimerase (DprE1), involved in the synthesis of cell wall-associated arabinan (59) (Figure 1). The results show that the probe retains a high specificity demonstrating recognition of *Mtb* over NTM, and recognition of live from dead bacteria. Although it was not tested in clinical samples, it is likely that it can detect live *Mtb* with high performance. The implementation of such probes in combination with cost-effective imaging devices could represent a major revolution in the diagnosis of TB.

In a recent study, Barr and co-workers reported a new procedure utilizing flow cytometry of mycobacteria double stained with Calcein-AM and SYBR-Gold, allowing differentiation of live from dead bacilli, as Calcein-AM becomes active after being esterified by live bacilli, while SYBR-Gold binds DNA (60). Such approach demonstrates detection of viable bacteria within 90 min, compared to the standard CFUs procedure, which takes days. Remarkably, only 60% of the detected live bacilli grew in colonies, indicating detection of an important proportion of VBNC bacteria. Despite the ease of use and the detection capacity, the application of this procedure in clinical samples remains to be tested. Moreover, the requirement of trained personnel for data interpretation may deter its exploitation in endemic settings. As mentioned for bacteriophages, the detection of live bacilli out of TB or subclinical TB spectrum states would be problematic due to low bacterial burden (Figure 2 and Table 1).

### Chemiluminescent probes

Luminescent detection of *Mtb* is a promising alternative to fluorescence because it is technically easier and only requires a simple (and inexpensive) luminometer, instead of advanced optics instruments. This characteristic makes luminescence practical for point-of-care use. Fast luminescent affordable sensor of Hip1 (FLASH) is a novel chemiluminescent probe based on the detection of the activity of the serine-protease Hip1 (61) (Figure 1). Hip1 [Hydrolase important for pathogenesis 1; also known as carboxylesterase A (CaeA); Rv2224c] is a *Mtb* protease involved in down regulation of the host’s immune response. It is associated to the cell envelope, thus being a good target for detection in clinical samples. Besides, it is specific for mycobacteria, not having homolog in the human genome. However, the potential cross-reactivity with NTM protein orthologs may dilute the performance of this probe. Nevertheless, FLASH is highly sensitive and can differentiate live from dead bacilli in sputum samples detecting as few as 4000 bacteria in 1 h (61). In addition, FLASH showed esterase activity in replicating and in hypoxia-induced non-replicating *Mtb* (62), indicating that it could be detected in non-replicating states. This fact, together to its link to the immune response, makes this procedure a promising tool to assess an incipient status in the TB spectrum. However, the necessity of targeting the physical bacillus could restricts the use of this probe to disease statuses with higher bacterial loads such as TB or subclinical TB (Figure 2 and Table 1).

### Mycobacterial extracellular vesicles

The new paradigm in cell-cell communication involving trafficking of extracellular vesicles (EVs) has revolutionized many of our concepts of cellular physiology. Research in this topic has boomed and today it is widely recognized that most forms of life produce EVs, suggesting an important evolutionarily conserved mechanism of intercellular signaling. While EVs are already a valuable diagnostic entity in other human diseases such as cancer, much work is needed to establish the procedures to isolate and properly identify these structures in infected individuals. We demonstrated that *Mtb* produces EVs *in vitro* and *in vivo* as part of a sophisticated mechanism to manipulate host cellular physiology and evade the host immune system (63). *Mtb* EVs (MEVs) have immunomodulatory properties *in vitro* and when administered to mice (63, 64), possess promising vaccine properties (65) and seem to be genetically regulated (66). An EV is a biological particle containing information about the cell that released it. This notion is particularly important for a cryptic microorganism like *Mtb*, whose physiology cannot be separated from that of its only host, the human being. Moreover, the fact that EVs are widely present in biological fluids, make them excellent candidates as biomarkers. Detection of EVs in the context of *Mtb* infection could theoretically be performed at all stages of the disease since no physical detection of the bacillus is required (Figure 2 and Table 1). However, it needs to be determined whether production of EVs occurs at early states of the disease. Nevertheless, proper detection of these structures would initially require immunocapture tools and highly sensitive reagents currently under development.

## Concluding remarks

One of the greatest concerns about TB is the risk of developing disease in the nearly two billion people who show evidence of past *Mtb* infection and are supposedly latently infected. The classical view of latency points to a disease state without clinical symptoms and no evidence of microbial growth. However, recent studies indicate that LTBI belongs to a TB-spectrum, and standard procedures, based on immune reactivity, could misdiagnose cured individuals with reactivity to past infection as latently infected. Most of what we know about latency in TB is based on inadequate *in vivo* and *in vitro* models relative to development of latency in humans. However, such models have provided key information about regulatory aspects of the adaptation of *Mtb* to non-replicating conditions. The integration of data derived from such models through bioinformatics and *in silico* approaches is allowing to define core genes and host-related molecules as surrogates of LTBI and generate predictive models of disease progression (67, 68).

Theoretically, the identification of live *Mtb* within the spectrum of early TB-infected individuals would help to define the population who may be at risk of developing TB. Acknowledging that both TST and IGRA assays are routinely used to define TB infection in humans but are unable to distinguish it from sustained responses to a cured TB infection, assays able to provide a direct or indirect measurement of live *Mtb* in early stages of the disease would represent a breakthrough in TB control. Due to the lack of symptoms at this end of the spectrum, another difficulty arises associated with the selection of an adequate clinical sample for detection. Ideally, the sampling should be minimally invasive, limiting the choice to saliva, urine, or blood as preferential samples, all of which typically have a low burden of bacilli, thus emphasizing the concomitant requirement of highly sensitive procedures.

Here, we have searched for diagnostic approaches, which specifically can measure host-independent parameters relying on the viability of *Mtb* (Table 1). Most of the methods examined support their enhanced ability to physically detect few bacilli and distinguish live from dead mycobacteria (MPT64, fluorescence probes or fluorobacteriophages). Although, this feature is ideal for a diagnostic approach, in the context of early TB infection it may represent an issue because of the low bacterial burden. In fact, those previously mentioned approaches have proven their feasibility in sputum samples from individuals with TB. Providing a good sensitivity and wide range of strain infectivity, the detection of viable bacilli using fluorobacteriophages is theoretically possible across the statuses within the TB spectrum. Moreover, the level of fluorescence would be related to the number of live bacilli present in each of them. On the other hand, targets of the fluorescent probes, which are based on synthesis of cell-wall components, would most probably be required when bacilli started active cell division, supposedly when establishing a subclinical form of the disease. A chemiluminescent probe based on the activity of Hip1 has shown the capacity to detect non-replicating bacilli. According to that, we can hypothesize that this procedure could be suitable for detection of bacilli related to early TB infectious statuses, including subclinical or incipient TB. Regarding the detection of MPT64, previous data suggested that this approach may not be reliable during silent infection, potentially limiting this method to subclinical or advanced TB. The amplification of pre-rRNA in a wide range of culture negative clinical samples (Table 1) showed its suitability for detection of viable bacilli, suggestive of a possible VBNC phenotype. Because of the link between pre-rRNA and metabolic activity, its detection represents not only a reliable marker of bacterial viability, but also an approach to reliably measure levels of live bacilli in the sample. Therefore, this procedure would be applicable along the spectrum of infection ranging from LTBI to TB. Another promising approach, currently under development, is the detection of *Mtb* EVs. The fact that EVs are widely present in biological fluids and are only made by live bacteria, make them excellent candidates as biomarkers of viability. The detection of EVs in the context of infectious diseases is still in its infancy. While EVs are already a valuable diagnostic entity in other human diseases such as cancer, much work is needed to establish the procedures to isolate and properly identify these structures in *Mtb*-infected individuals.

The combination of these approaches with improvements in sample processing and novel analytical platforms, as well as the development of *in silico* approaches, including artificial intelligence (AI) and bioinformatics could help to initially distinguish TB from LTBI (69) and eventually allow more precise definition of risk of LTBI individuals to develop TB disease.

## Author contributions

MG and RP-R conceived, designed the study, and revised the manuscript for submission. SA, MG, and RP-R performed the search for relevant literature. BW and JA revised the manuscript and made contributions to specific sections of the manuscript. All authors approved the manuscript.
